# Fornical and nonfornical projections from the rat hippocampal formation to the anterior thalamic nuclei

**DOI:** 10.1002/hipo.22421

**Published:** 2015-03-03

**Authors:** Christopher M. Dillingham, Jonathan T. Erichsen, Shane M. O'Mara, John P. Aggleton, Seralynne D. Vann

**Affiliations:** ^1^ School of Psychology, Cardiff UniversityCardiffUnited Kingdom; ^2^ School of Optometry and Vision Sciences, Cardiff UniversityCardiffUnited Kingdom; ^3^ Trinity College Institute of Neuroscience, Trinity College DublinDublinIreland

**Keywords:** anatomy, fornix, hippocampus, memory, postsubiculum, subiculum

## Abstract

The hippocampal formation and anterior thalamic nuclei form part of an interconnected network thought to support memory. A central pathway in this mnemonic network comprises the direct projections from the hippocampal formation to the anterior thalamic nuclei, projections that, in the primate brain, originate in the subicular cortices to reach the anterior thalamic nuclei by way of the fornix. In the rat brain, additional pathways involving the internal capsule have been described, linking the dorsal subiculum to the anteromedial thalamic nucleus, as well as the postsubiculum to the anterodorsal thalamic nucleus. Confirming such pathways is essential in order to appreciate how information is transferred from the hippocampal formation to the anterior thalamus and how it may be disrupted by fornix pathology. Accordingly, in the present study, pathway tracers were injected into the anterior thalamic nuclei and the dorsal subiculum of rats with fornix lesions. Contrary to previous descriptions, projections from the subiculum to the anteromedial thalamic nucleus overwhelmingly relied on the fornix. Dorsal subiculum projections to the majority of the anteroventral nucleus also predominantly relied on the fornix, although postsubicular inputs to the lateral dorsal part of the anteroventral nucleus, as well as to the anterodorsal and laterodorsal thalamic nuclei, largely involved a nonfornical pathway, via the internal capsule. © 2015 The Authors Hippocampus Published by Wiley Periodicals, Inc.

## INTRODUCTION

Two principal brain regions implicated in anterograde amnesia, and hence episodic memory, are the medial temporal lobe and the medial diencephalon. Within the respective regions, attention has repeatedly focused on the hippocampus and the anterior thalamic nuclei, along with their reciprocal interactions (Barbizet, 1963; Delay and Brion, 1969; Parker and Gaffan, 1997; Aggleton and Brown, 1999). Both animal lesion studies and clinical data have, for example, shown how anterior thalamic damage or disconnection can mimic many of the effects of hippocampal damage, i.e., causing memory loss in humans and spatial deficits in rodents (e.g., Aggleton and Sahgal, 1993; Harding et al., 2000; Van der Werf et al., 2003; Tsivilis et al., 2008; Wolff et al., 2008; Vann, 2010; Carlesimo et al., 2011). More direct evidence for their functional interdependence has come from crossed‐lesion disconnection studies in rats (Warburton et al., 2001; Henry et al., 2004). For these reasons, it is valuable to detail the precise manner by which the hippocampus projects to the anterior thalamic nuclei.

The anterior thalamic nuclei do not receive direct inputs from the CA fields of the hippocampus. Instead, in both rodents and primates, the dense anterior thalamic inputs arise from the subicular cortices (Swanson and Cowan, 1975; Meibach and Siegel, 1977a; Rosene and Van Hoesen, 1977; Sikes et al., 1977; Krayniak et al., 1979; Saunders et al., 2005). Many of these subicular projections join the postcommissural component of the fornix (Chronister et al., 1975; Swanson and Cowan, 1975; Poletti and Cresswell 1977; Krayniak et al., 1979; Aggleton et al., 1986; Saunders and Aggleton, 2007). The postcommissural fornix also contains the hippocampal projections to the mammillary bodies (Swanson and Cowan, 1975; Poletti and Creswell, 1977; Aggleton et al., 2005), which, in turn, relay to the anterior thalamic nuclei. Consequently, these fornical interconnections have been of particular interest for understanding how fornix damage may disrupt memory in humans (Gaffan and Gaffan, 1991; Aggleton et al., 2000; Tsivilis et al., 2008), as well as in non‐human primates and rats (e.g., Murray et al., 1989; Cassel et al., 1997; Bussey et al., 2000; Aggleton and Brown, 2002).

**Figure 1:**
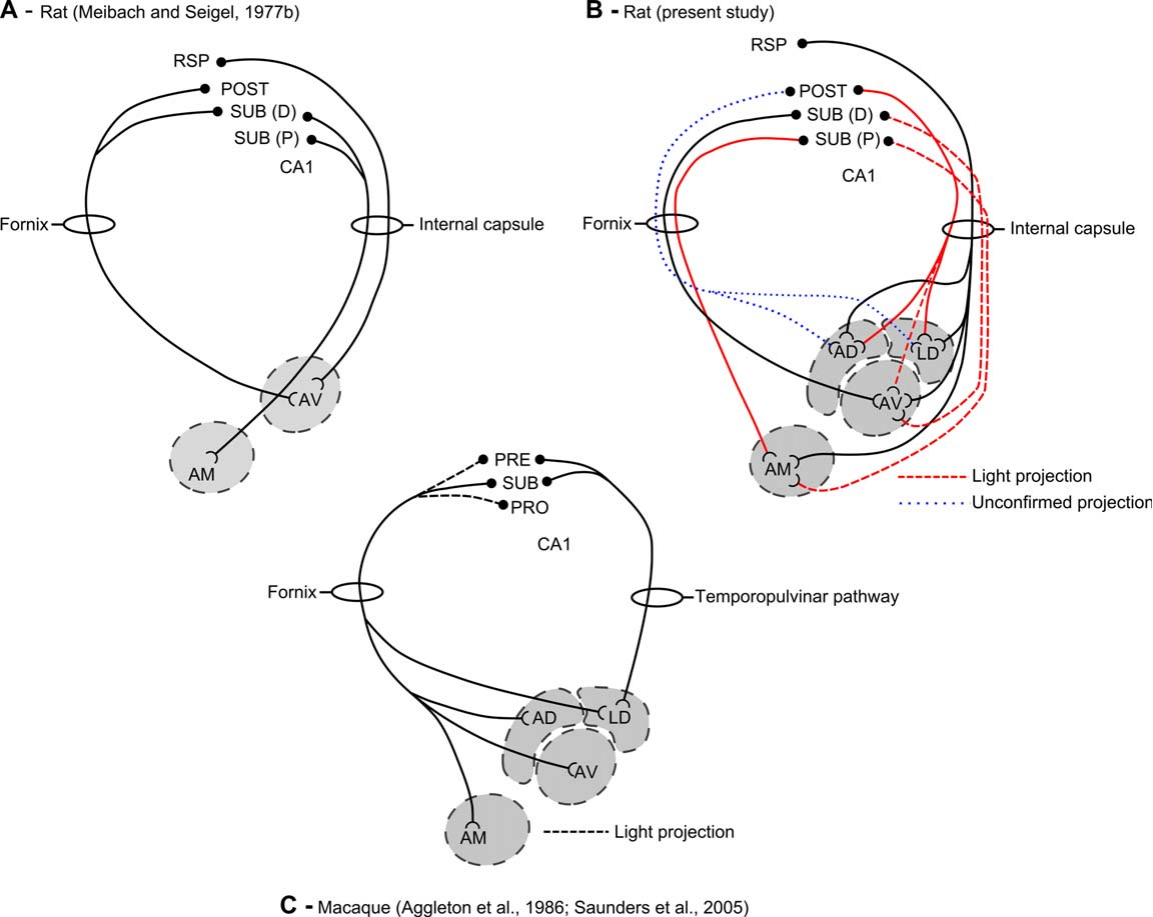
Schematic diagrams showing the purported fornical and nonfornical routes from the hippocampus to the anterior thalamic nuclei. A. The various pathways in the rat brain described by Meibach and Siegel (1977b). B. The pathways shown by the present study. In this schematic, solid red pathways represent additional information or amendments to Meibach and Siegel (1977b). The figure includes the light nonfornical input to the dorsolateral anteroventral thalamic nucleus along with the apparent light nonfornical input to the anteromedial thalamic nucleus (long‐dashed red pathways). There remains the possibility that some efferents from the postsubiculum also join the fornix to reach the anterodorsal and laterodorsal thalamic nuclei (short‐dashed blue pathways). C. The corresponding pathways in the monkey (macaque) brain (from Aggleton et al., 1986 and Saunders et al., 2005). AD, anterodorsal thalamic nucleus; AM, anteromedial thalamic nucleus; AV, anteroventral thalamic nucleus; CA1, hippocampal field CA1; LD, laterodorsal thalamic nucleus; POST, postsubiculum; PRE, presubiculum; PRO, prosubiculum; RSP, retrosplenial cortex; SUB, subiculum; SUB (D), distal subiculum; SUB (P) proximal subiculum. [Color figure can be viewed in the online issue, which is available at wileyonlinelibrary.com.]

A potential, major species difference in these pathways was reported in a study of the rat brain (Meibach and Siegel, 1977b). That study (see Fig. 1A) described how the subicular inputs to the anteromedial thalamic nucleus almost exclusively use a pathway via the internal capsule, i.e., are nonfornical (but see Swanson and Cowan, 1977; Swanson et al., 1987). In contrast, the projections to the rat anteroventral thalamic nucleus still depend on the fornix (Meibach and Siegel, 1977b). This is a striking result as it appears very different from that found in the monkey brain, where the hippocampal inputs to both the anteromedial and anteroventral thalamic nuclei rely exclusively on the fornix (see Fig. 1C; Aggleton et al., 1986; Saunders et al., 2005).

In the tracer study by Meibach and Siegel (1977b), anterograde and retrograde tracers were injected into the subicular cortices and the thalamus, respectively. A potential limitation, however, is that their data came solely from intact animals, i.e., they did not map hippocampal projections after the fornix had been severed. A further concern is that other, subsequent reports often depict the subicular inputs to the anterior thalamic nuclei, including those to the anteromedial nucleus, as principally relying on the fornix (e.g., Swanson et al., 1987). In view of this uncertainty, the present study re‐examined the pathways taken by the hippocampal formation to the anterior thalamus by studying rats with fornix lesions. Tracer injections were made into the anterior thalamic nuclei (retrograde) or hippocampal formation (anterograde). The thalamic retrograde tracer injections chiefly targeted the anteromedial thalamic nucleus, thought to be the principal termination site for the nonfornical inputs to the anterior thalamus (Meibach and Siegel, 1977b). In addition, tracer injections were directed at the anteroventral and laterodorsal thalamic nuclei. Despite its lack of mammillary body inputs, the laterodorsal nucleus is regarded by some as one of the anterior thalamic group of nuclei (Jones, 1985; Bentivoglio et al., 1993). The laterodorsal thalamic nucleus also receives direct hippocampal inputs from the subicular cortices, but in the rat, these projections arise from the postsubiculum and are thought to reach the thalamus largely via the internal capsule (van Groen and Wyss, 1990b).

## MATERIALS AND METHODS

**Figure 2:**
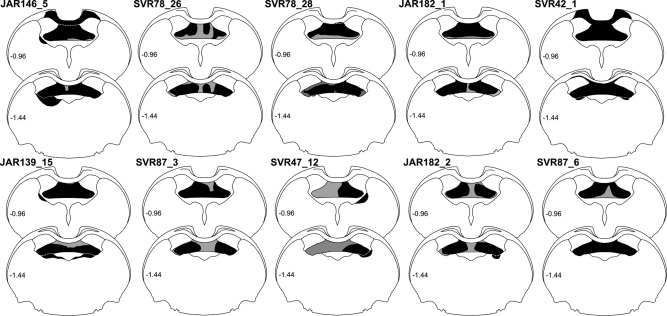
Coronal sections showing the extent of the fornix lesions (in black) in the 10 reported cases. Any additional thalamic damage is also indicated. The numbers refer to the location of the sections with respect to bregma (see Paxinos and Watson, 2005).

A total of 18 male Lister‐Hooded rats (Harlan, UK), weighing 290 to 322 g at the time of surgery (Fig. 2, Table 1), were used in the study. Of these, 10 animals received bilateral fornix lesions and one (case SVR47_12) received a unilateral fornix lesion. In two of these animals, Fast Blue (Polysciences Inc. Eppelheim, Germany) was injected unilaterally into the anteromedial thalamic nucleus. One (case SVR42_1) received a unilateral injection of Diamidino Yellow into the laterodorsal thalamic nucleus. In four animals, two fluorescent retrograde tracers, Fast Blue and Diamidino Yellow (Sigma‐Aldrich, Gillingham, UK), were injected into one or more of the anterior thalamic nuclei. In two animals (cases SVR87_6 and SVR78_28), multiple anterograde injections of the pathway tracer horseradish peroxidase conjugated with wheat germ agglutinin (WGA‐HRP; Vector Laboratories, Peterborough, UK) were made unilaterally into the postsubiculum. In two further animals (JAR182_1, JAR182_2), anterograde tracer injections were targeted at the subiculum along with retrograde tracers in the anterior thalamus. In case JAR182_1, multiple injections of BDA (Life Technologies Ltd, Paisley, UK) were directed at the dorsal subiculum in one hemisphere, with comparable injections of Fluororuby (Life Technologies Ltd, Paisley, UK) into the dorsal subiculum in the other hemisphere, as well as an injection of Fast Blue into the right anteroventral thalamic nucleus. In case JAR182_2, multiple, bilateral injections of Fluororuby were targeted at the proximal dorsal subiculum, again combined with an injection of Fast Blue into the anteroventral thalamic nucleus. Finally, in seven rats without fornix lesions, retrograde (four rats) and anterograde (three rats) tracer injections were made into the anterior thalamus and subicular cortices, respectively. All experiments were carried out in accordance with the UK Animals (Scientific Procedures) Act, 1986 and associated guidelines, and approved by the ethical committees of Cardiff University.

**Table 1:** Summary Table Showing Tracer Type and Location of Tracer Injection

Case #	Tracer		AM		LD		AV		AD
	Left	Right	Left	Right	Left	Right	Left	Right	
Retrograde tracer injections (with fornix lesions)									
SVR78_28	FB		+						+
SVR87_3	FB		+						+
JAR146_5	FB	FB	+	+			+	+	+
SVR42_1	FB/DY	FB/DY	+ (FB)	+ (FB)	+ (DY)	+ (DY)	+	+	+
JAR139_15	FB	DY	+	+				+	+
SVR78_26	FB		+						+
SVR47_12	FB	FB	+	+			+	+	+
JAR182_1		FB						+	
JAR182_2		FB						+	
SVR78_26	FB		+						
Retrograde control cases (no fornix lesion)									
SVR88_5	FB						+		
SVR88_6	FB						+		
SVR77_6	FB		+						
SVR75_9	FB				+				

Case #	Tracer		SUB (P)		SUB (D)		POST	
	Left	Right	Left	Right	Left	Right	Left	Right
Anterograde tracer injections (with fornix lesions)								
SVR78_28	WGA‐HRP				*		+	
SVR87_6	WGA‐HRP				*		+	
JAR182_1	BDA	FR	+	+	+	+		
JAR182_2	FR	FR	+	+	+	+		
Anterograde control cases (no fornix lesion)								
JAR178_1	WGA‐HRP	WGA‐HRP	+	+	+	+	+	+
JAR182_3	FR	BDA	+	+	+	+		
JAR182_4	FR	BDA	+	+	+	+		

Upper: cases that combined fornix lesions with retrograde tracer injections into the anterior thalamus. Upper middle: control cases (no fornix lesion) that received retrograde tracer injections. Lower middle: cases that combined fornix lesions with anterograde tracer injections into the subicular cortices. Lower: control cases (no fornix lesion) that received anterograde tracer injections into the subicular cortices. Plus symbols (+) indicate tracer uptake.

AM, anteromedial thalamic nucleus; AD, anterodorsal thalamic nucleus; AV, anteroventral thalamic nucleus; FB, Fast Blue; DY, Diamidino Yellow; LD, laterodorsal thalamic nucleus; POST, postsubiculum; SUB, subiculum; SUB (D), distal subiculum; SUB (P) proximal subiculum.

Before surgery, four of the eighteen animals were deeply anesthetized by intraperitoneal injection of sodium pentobarbital (60 mg/kg pentobarbital sodium salt; Sigma‐Aldrich, UK). For the remaining animals, surgery was performed under an isoflurane‐oxygen mixture (1.5–2.5% isoflurane). In all cases**,** a subcutaneous injection of Metacam (1 mg/kg body weight, Boehringer Ingelheim, Germany) was given during surgery to provide post‐operative analgesia.

### Fornix Lesions

The fornix lesions were made by radiofrequency using a thermocouple radiofrequency electrode (0.3 mm active tip length, 0.25 mm diameter; Diros Technology Inc., Ontario, Canada). The electrode was lowered vertically, and the tip temperature was raised to 70–74°C for 60 s using an OWL Universal RF System URF‐3AP lesion maker (Diros Technology Inc. Ontario, Canada). Two lesions were made in each hemisphere at the same anteroposterior (AP) level. The stereotaxic coordinates from bregma were: anteroposterior (AP) +0.0, lateromedial (LM) ±0.5–0.7, dorsoventral (DV) −4.7; LM ± 1.7, DV −4.8. In all cases, the nose bar was set at +5.0 mm.

### Tracer Selection

Two retrograde tracers (Fast Blue and Diamidino Yellow), widely used and regarded as highly sensitive and robust (Lanciego and Wouterlood, 2011), were used to visualise anterior thalamic afferents. Both tracers are transported retrogradely and monosynaptically, and result solely in cell body label, i.e. no fiber tract or neuropil label. As such, the description of label resulting from injections of Fast Blue and Diamidino Yellow in the results section below applies only to cell body fluorescence. The tracer WGA‐HRP can be transported both anterogradely and retrogradely, typically in equal measure, making it important to distinguish the nature of any label. Terminal label is seen as aggregations of fine particles while retrograde label is confined to the cell somas. As tracts are also readily visualised with WGA‐HRP, it is important to appreciate that a labeled tract may contain anterograde label, retrograde label, or both. Although WGA‐HRP can be transported trans‐synaptically (Itaya, 1987), the survival time used for the cases described here (2 days) means that trans‐synaptic transport is unlikely to have taken place (Itaya, 1987). The limitations of WGA‐HRP for tract determination were compensated by two additional tracers BDA (3,000 MW) and Fluororuby (rhodamine‐dextran‐amine). Although both of these tracers can be transported bidirectionally, axonal fiber label only takes place in the anterograde direction. While it has been reported that anterograde transport can take place subsequent to retrograde transport (i.e., via axon collaterals; Reiner et al., 2000), optimising the parameters used, i.e. the molecular weight (3,000 MW) and the pH (7.4) of the delivery vehicle, the relative proportion of retrograde to anterograde transport can be reduced. In the results section below, descriptions of the pattern of label following BDA and Fluororuby tracer injections are defined as either fiber/terminal label or cell body label in order to differentiate between anterograde or retrograde transport, respectively.

### Tracer Injections

Tracer injections were made using either a 0.5 or 1.0 µL Hamilton syringe (Hamilton, Bonaduz, Switzerland). The retrograde tracers, Fast Blue (Polysciences Inc. Eppelheim, Germany) and Diamidino Yellow, were made up as 3% solutions in sterile phosphate buffer saline (PBS), while WGA‐HRP was made up to a concentration of 40 mg/mL. Biotinylated dextran amine and Fluororuby were made up as 10% solutions in sterile, distilled water and 0.1M PBS, respectively. Typically, injection volumes at each site were 0.03 to 0.04 µL for Fast Blue, 0.05 to 0.06 µL for Diamidino Yellow, 0.04 to 0.05 µL for WGA‐HRP and 0.06 to 0.08 µL for BDA and Fluororuby. The pressure injections were made over the course of 10 min with the needle left in situ before injection for 5 min. Injections into the anterior thalamic nuclei were centered around the co‐ordinates (from bregma): AP −0.2, LM ±0.7, DV −7.0, and AP −0.2, LM ±1.5, DV −6.2 (based upon Paxinos and Watson, 2005). Laterodorsal thalamic nucleus injections were made at AP −0.8, LM ±2.2, DV −6.4. Injections of WGA‐HRP into the postsubiculum were made in three sites along its anterior‐posterior axis: caudal; AP −5.3, LM ±4.6, DV −8.1, intermediate; AP −5.0, LM ±3.5, DV −6.5 and rostral; AP −4.4, LM ±2.4, DV −5.8. Injections of BDA into the dorsal subiculum (e.g. case JAR182_1) were, again, made at three sites along the anterior‐posterior axis: caudal; AP −5.3, LM ±4.9, DV −8.3, intermediate; AP −5.0, LM ±3.8, DV −6.7 and rostral; AP −4.4, LM ±2.9, DV −5.8.

### Perfusion/Fixation

Post‐surgery, the skin was sutured, and animals received a 5‐mL subcutaneous injection of 5% glucose in 0.9% saline (Baxter Healthcare Ltd, Norfolk, UK). Clindamycin hydrochloride antibiotic powder (Pharmacia Ltd, Sandwich, UK) was applied topically. Animals were then allowed to recover in a thermostatically controlled container before being returned to their home cage with food and water available ad libitum. Following a postoperative period of 2 to 4 days that depended on the tracer (WGA‐HRP: 2 days; Fast Blue/BDA/Fluororuby: 4 days), the animals were deeply anesthetized with sodium pentobarbital (Euthatal, Merial, Harlow, UK). All rats were initially perfused intracardially with 0.1M PBS (pH 7.4) at room temperature. In those animals that received injections of fluorescent tracer or BDA, the PBS was followed by 4% paraformaldehyde in 0.1M PBS at ∼4°C, while those animals that received injections of WGA‐HRP into the dorsal subiculum received a fixative made up of 1.5% paraformaldehyde and 1.5 to 2% gluteraldehyde, again at ∼4°C.

### Histology

#### Fluorescent tracer injections

Brains were removed and placed in the dark for 4 h in fixative and then transferred to a 25% sucrose solution in 0.1M PBS for 24 h in the dark to cyroprotect the tissue before cutting. Brains were placed on a freezing platform, and 40 µm coronal sections were cut on a sledge microtome (Leica 1400). For those cases in which fluorescent tracers were injected into anterior thalamic nuclei (Fast Blue, Diamidino Yellow) or the dorsal subiculum (Fluororuby), two “1‐in‐3” series of sections were mounted directly onto gelatin‐subbed slides and then allowed to dry in the dark at room temperature. A third series was stained with cresyl violet in order to assess the extent of the fornix lesions and to help localise the various injection sites.

#### Horseradish peroxidase‐conjugated wheatgerm agglutinin (WGA‐HRP)

Brains were removed and postfixed for 4 h in a 1.5% paraformaldehyde and 1.5 to 2% glutaraldehyde in 0.1M PBS solution and then transferred to a 25% sucrose solution in 0.1M PBS for 24 h in the dark to cyroprotect the tissue before cutting. Brains were placed on a freezing platform, and 40 µm coronal sections were cut on a sledge microtome (Leica 1400). One “1‐in‐3” series was mounted directly onto gelatin‐subbed slides while the remaining series were collected in 0.1M PBS (pH 6.0) for the subsequent 3,3′,5,5′‐tetramethylbenzidine reaction (TMB) for visualization of anterogradely transported WGA‐HRP. For the TMB reaction, sections were incubated, with agitation, in a fresh 0.1M phosphate buffer (PB, pH 6.0) solution before being incubated at room temperature in a solution containing 0.25% ammonium molybdate in 0.1M PB and 0.002% 3,3′,5,5′‐tetramethylbenzidine, dissolved in 100% ethanol, for 30 min. Following incubation, a 1% hydrogen peroxide solution in distilled water was added in three stages, at 30 min intervals, until the final concentration of hydrogen peroxide was 0.3%. Sections were then incubated in the same solution overnight at 4°C. The TMB reaction precipitate was stabilized through subsequent incubation of sections in a 5% ammonium molybdate solution in 0.1M PB (Ph 6.0) for 30 min according to the methodology of Marfurt et al. (1988). Following incubation, sections were washed in 0.1M PB (pH 6.0) before being mounted on gelatin‐subbed slides and left to dry overnight at room temperature. The sections were then dehydrated in ascending alcohols and coverslipped with DPX mountant (Sigma‐Aldridge, Gillingham, UK).

#### Biotinylated dextran amine (BDA)

Brains were removed and postfixed in a 4% paraformaldehyde solution in 0.1M PBS and cryoprotected as above. In all cases, the BDA injections were made in combination with the fluorescent tracer Fluororuby in the contralateral hemisphere. For this tissue, a “1‐in‐4” series was mounted directly for fluorescence imaging and one for cresyl violet staining. Another series was collected free floating in 0.1M PBS before being washed three times, for 10 min each, in 0.1M PBST. Sections were incubated in the Vectastain ABC solution (Vector Labs, Peterborough, UK) for 2 h, then washed in PBST twice for 10 min each followed by a further three washes in 0.1M PBS. Sections were then reacted with diamino benzidine (DAB; Vector Labs, Peterborough, UK) and intensified with nickel after which they were mounted, dried and coverslipped, as described above.

#### Microscopy and imaging

A Leica DM5000B microscope with a Leica DFC310FX digital camera and Leica Application Suite image acquisition software were used for brightfield and fluorescence microscopy.

## RESULTS

In addition to describing any retrogradely transported label in the hippocampus, the presence of retrograde cell body label in the mammillary bodies helped to confirm the effective location of the thalamic tracer injections. The projections from the medial mammillary nucleus to the anterior thalamic nuclei are ipsilateral, with pars medialis providing the majority of inputs to the anteromedial thalamic nucleus, while pars lateralis largely projects to the anteroventral nucleus (e.g,. Takeuchi et al., 1985; Shibata, 1992; Hopkins, 2007). In contrast, the lateral mammillary nucleus projects bilaterally to the anterodorsal thalamic nucleus.

The results include details of comparable cases that have no fornix lesions, i.e., are intact, or have significant amounts of tract sparing. These cases make it easier to appreciate any changes in labeling related to fornix damage. Multiple injection cases are described (see Table 1). The rationale was to determine the reliability of the findings, despite the variations in the tracer being used and the different targets (retrograde: anteromedial, anteroventral, and laterodorsal nuclei; anterograde: postsubiculum and dorsal subiculum). A further factor was the appreciation that the results differed markedly from those of Meibach and Siegel (1977b), underscoring the need to describe multiple cases. Other considerations were the inevitable variability in the fornix lesions (Fig. 2) and the extent to which the surgeries could potentially involve other pathways.

### Group 1—Retrograde Tracer Injections Into the Anteromedial Thalamic Nucleus With Extensive Fornix Lesions (Fornix Lesion Cases JAR146_5, JAR139_15, and SVR78_26)

In two of these cases, extensive lesions of the fornix were combined with bilateral injections of retrograde tracer into the anteromedial thalamic nucleus. In case JAR146_5, Fast Blue was injected bilaterally, while in case JAR139_15, Fast Blue and Diamidino Yellow were injected into the left and right hemispheres, respectively. In both cases, given the extent of the fornix lesions, some subcortical (dorsal thalamic) damage resulted. In both cases, subicular label was virtually absent (<2 cells/section).

**Figure 3:**
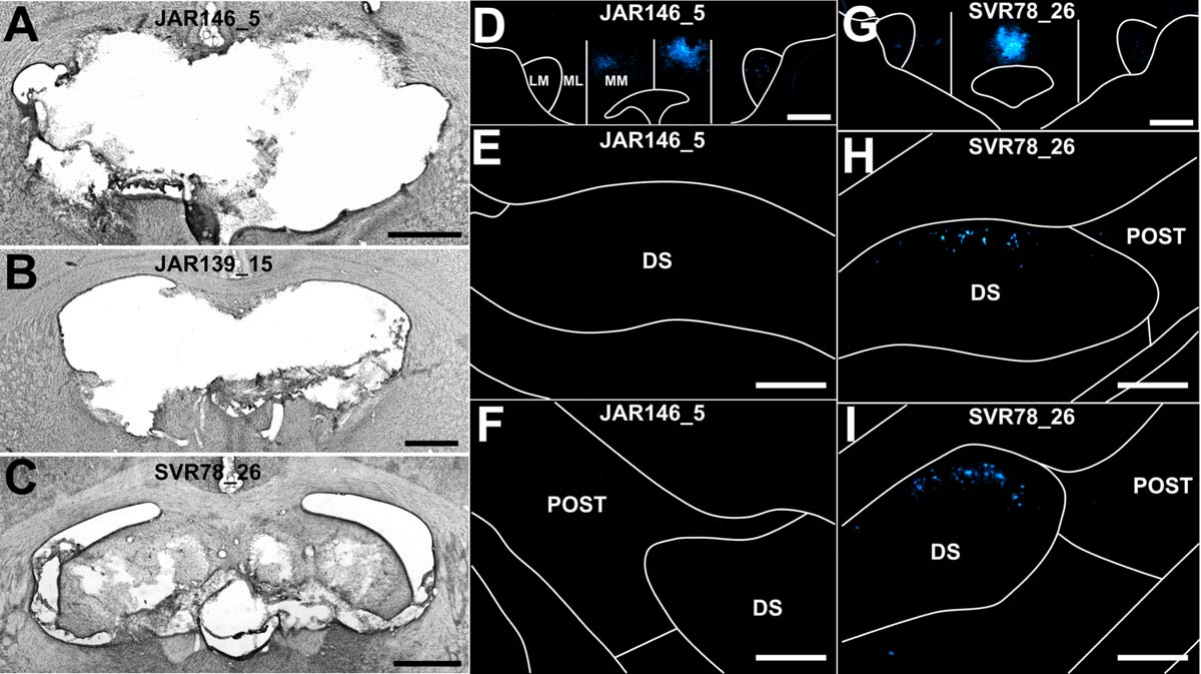
Brightfield and fluorescent photomicrographs of three fornix lesion cases, two of which had extensive tract lesions (JAR146_5 and JAR139_15), while case (SVR78_26) had some sparing at the midline. The fornix lesion surgeries were combined with bilateral (JAR146_5 and JAR139_15) or unilateral (SVR78_26) Fast Blue injections into the anteromedial thalamic nucleus. A–C. Brightfield photomicrographs of the fornix lesions that depict the most damaged region of the fornix. D–F. Fluorescent photomicrographs of retrograde label (in a case with an extensive fornix lesion; JAR146_5) in pars lateralis of the medial mammillary body nucleus (D) yet showing an absence of retrograde label in the dorsal subiculum at both rostral (E) and at caudal anteroposterior levels (F); G–I. Fluorescent photomicrographs of a case with a partial fornix lesion (SVR78_26) in which the retrograde label is confined to pars medialis of the medial mammillary body nucleus (G) as well as light retrograde label in rostral (H) and caudal (I) levels of the dorsal subiculum. CC, corpus callosum; DS, dorsal subiculum; LM, lateral mammillary body nucleus; ML, pars lateralis of the medial mammillary body nucleus; MM, pars medialis of the medial mammillary body nucleus; POST, postsubiculum. Scale bars: A–D and G = 1,000 µm; E, F and H, I = 250 µm. [Color figure can be viewed in the online issue, which is available at wileyonlinelibrary.com.]

In case JAR146_5, attention focussed on the distribution of label in the right hemisphere for two reasons. In this hemisphere, the fornix lesion appeared complete while the thalamus was spared (Figs. 2 and 3A). Furthermore, there was abundant Fast Blue cell body label within pars medialis of the right medial mammillary nucleus consistent with uptake in the right anteromedial nucleus (Fig. 3D). The much lighter retrograde cell body label within pars lateralis of the medial mammillary nucleus suggested light, additional uptake from within the anteroventral thalamic nucleus. A few bilateral retrogradely labeled Fast Blue labeled cells were present in the lateral mammillary nucleus, potentially reflecting tracer uptake by the anterodorsal nucleus. Within the hippocampal formation, no retrogradely labeled Fast Blue cells were observed in the right dorsal subiculum (Figs. 3E,F) and very few (<2 cells per section) were present in the postsubiculum of the right hemisphere (Fig. 3F).

In the left hemisphere of case JAR146_5, there was an abundance of retrogradely labeled cell bodies in the pars medialis subdivision of the medial mammillary nucleus with relatively few retrogradely labeled cell bodies in pars lateralis of the medial mammillary nucleus. The fornix lesion extended ventrally to reach the upper thalamus, including the dorsal anterodorsal nucleus, dorsal anteroventral nucleus, and part of the stria terminalis (Fig. 3A). In the same hemisphere, Fast Blue retrogradely labeled cell bodies were again almost completely absent from the hippocampal formation. No labeled cells were present in the left proximal subiculum, while the very few retrogradely labeled cells that were present (<2 cells/section) were predominantly located in the postsubiculum but occasionally also in the distal subiculum. No retrogradely labeled cell bodies were present in the ventral subiculum in either hemisphere.

In case JAR139_15 uptake from the bilateral injections in the anteromedial thalamic nuclei was reflected by a dense plexus of retrogradely labeled Fast Blue cells in pars medialis of the medial mammillary nucleus in the left hemisphere and retrograde Diamidino Yellow cell body label, albeit weaker, in the corresponding nucleus of the right hemisphere. Weak retrograde Diamidino Yellow label was also present in cell bodies within pars lateralis of the medial mammillary nucleus, consistent with uptake by the neighboring anteroventral thalamic nucleus. Bilateral retrogradely labeled Fast Blue and Diamidino Yellow cells were also found in the lateral mammillary nucleus, although for the latter tracer, there were considerably fewer cells. While the fornix lesion just touched the dorsal edge of the anteroventral nucleus in both hemispheres, tracts such as the stria terminalis were intact (Figs. 2 and 3B).

Retrograde Fast Blue and Diamidino Yellow cell body label in case JAR139_15 was virtually absent in both proximal and distal regions of the dorsal subiculum in both hemispheres, however those labeled cells that were present (<2 cells/section) were confined to distal regions. There was extensive retrograde Fast Blue cell body label in the postsubiculum of the left hemisphere consistent with Fast Blue uptake by the anterodorsal thalamic nucleus in the left hemisphere. Only sparse Diamidino Yellow cell body label was present in the right postsubiculum, consistent with the lower density of retrogradely labeled cell bodies present in the lateral mammillary nuclei. Again, no retrogradely labeled cell bodies were present in the ventral subiculum of either hemisphere.

In a third case (SVR78_26), the bilateral fornix lesion was incomplete with midline sparing, as well as sparing within the dorsal and lateral extent of the fimbria (Figs. 2 and 3C). An injection of Fast Blue was centered in the anteromedial thalamic nucleus. Retrograde cell body label in the mammillary bodies was virtually confined to pars medialis of the medial mammillary body nucleus (Fig. 3G), except for a very sparse cluster of retrogradely labeled cells bilaterally in the lateral mammillary nuclei, a pattern consistent with primary uptake by the anteromedial thalamic nucleus, i.e., largely exclusive of neighboring nuclei. Appreciable retrograde cell body label was present in the hippocampus, predominantly in the proximal dorsal subiculum in both hemispheres (20–40 cells/section; Figs. 3H,I). Only a few retrogradely labeled cells were present in the distal subiculum and in the ipsilateral postsubiculum (<3 cells/section; Figs. 3H,I), consistent with the primary injection locus in the anteromedial nucleus. Finally, no retrograde label was observed in cell bodies of the ventral subiculum in either hemisphere.

### Group 2—Retrograde Tracer Injections Into the Anteromedial Thalamic Nucleus With Selective Lesions of the Fornix (Fornix Lesion Cases SVR87_3, SVR78_28, and SVR47_12)

**Figure 4:**
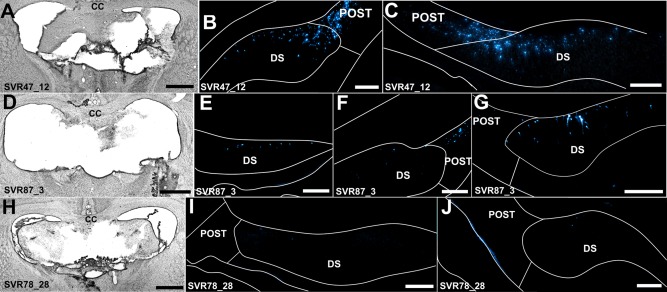
Brightfield and fluorescent photomicrographs of three fornix lesion cases that received injections of Fast Blue in the anteromedial thalamic nucleus. Case SVR47_12 received an incomplete unilateral (right hemisphere) lesion of the fornix (A) and bilateral injections of Fast Blue into the anteromedial thalamic nucleus, with additional uptake by the anteroventral thalamic nucleus. Resulting retrograde label in the dorsal subiculum and postsubiculum (B, C) was considerable in both hemispheres. The fornix lesion in SVR87_3 (D) was extensive but with some limited dorsomedial sparing in the right hemisphere. The Fast Blue injection site was centered in the left anteromedial thalamic nucleus with additional uptake by the anterodorsal thalamic nucleus. In the left hemisphere, retrograde label in the dorsal subiculum was weak (<10 cells/section; E, F) while dense label was present in the postsubiculum (F); retrograde label in the dorsal subiculum of the right hemisphere (G) was denser than that of the left (E, F), consistent with the fornix sparing described; Case SVR78_28 received an extensive lesion with only a small amount of lateral fimbria sparing (H) combined with an injection of Fast Blue into the anteromedial thalamic nucleus. Retrograde label in the dorsal subiculum (I, J) was virtually absent (<2 cells/section). Abbreviations: CC, corpus callosum; DS, dorsal subiculum; POST, postsubiculum. Scale bars: A, D and H = 1,000 µm; B, C, E, G, and I, J = 250 µm. [Color figure can be viewed in the online issue, which is available at wileyonlinelibrary.com.]

In two cases, unilateral injections of Fast Blue were made into the left anteromedial thalamic nucleus following bilateral lesions of the fornix (SVR87_3, SVR78_28). In case SVR87_ 3, the fornix lesion in the same hemisphere appeared complete (Figs. 2 and 4D). In the right hemisphere, the lesion was almost complete, although a small section of tissue in the dorsomedial part of the dorsal fornix was spared (Figs. 2 and 4D). In case SVR78_28, the fornix lesion was again extensive, involving most of the tract but with some sparing in the ventral‐most strip of tissue (Figs. 2 and 4H). In both cases, the dorsal thalamus was intact.

In both cases, abundant retrograde cell body label was confined to pars medialis of the ipsilateral medial mammillary nucleus, consistent with tracer uptake by the anteromedial thalamic nucleus. Additional retrograde cell body label was present bilaterally in the lateral mammillary nuclei, consistent with uptake by the anterodorsal thalamic nucleus. Such label was present in both cases, but was only very sparse in case SVR78_28.

In case SVR87_3, an appreciable number of retrogradely labeled cell bodies were present bilaterally throughout the postsubiculum (Fig. 4F). This label is indicative of tracer uptake by the anterodorsal thalamic nucleus, and subsequent transport via a nonfornical pathway. Some retrogradely labeled cells were also present bilaterally in the rostral‐most parts of the dorsal subiculum (6–10 cells/section; Fig. 4E), but more caudally this label in the left dorsal subiculum disappeared (Fig. 4F). Contralateral to the injection site, in the right dorsal subiculum, a small pocket of 10 to 15 retrogradely labeled cells per section was also present within the dorsal subiculum (Fig. 4G). The presence of this pocket of cells in the contralateral hemisphere corresponds to the observed partial sparing in the dorsomedial fornix of the right hemisphere (Fig. 4D), i.e., the region of the fornix that carries inputs to the anterior thalamus.

Similarly, in case SVR78_28, a few retrogradely labeled cells were present in the right dorsal subiculum of either hemisphere. These few cells were predominantly situated ipsilateral to the injection in the caudal, proximal subiculum (<5 cells/section; Figs. 4I,J), corresponding topographically to cells projecting to the anteromedial thalamic nuclei. In addition, a very sparse population of retrogradely labeled cell bodies was present in the ipsilateral postsubiculum (<3 cells/hemisphere; Figs. 4I,J), corresponding to weak tracer uptake by the anterodorsal thalamic nucleus and subsequent retrograde transport via a nonfornical pathway. In both SVR87_3 and SVR78_28, a small number of retrogradely labeled cells were present in the ventral subiculum, ipsilateral to the Fast Blue injection (5–10 cells/section).

In a further case (SVR47_12), there was partial sparing of the medial fornix in the right hemisphere, along with complete sparing of the fornix in the left hemisphere (Figs. 2 and 4A). The Fast Blue injections involved the anteromedial and anteroventral thalamic nuclei in both hemispheres. As a consequence, bilateral Fast Blue labeled cells were present in both pars medialis and pars lateralis of the medial mammillary nucleus. Bilateral cell body label was also present in the lateral mammillary nucleus, consistent with uptake by the anterodorsal thalamic nuclei in one or both hemispheres. In the left (intact) hemisphere, a substantial number of Fast Blue labeled cells were present throughout the deep layers of the dorsal subiculum, i.e., in both proximal and distal regions (>50 cells per section; Fig. 4B). In the right hemisphere, i.e., where there was a partial fornix lesion (Fig. 4A), retrograde cell body label was present in the most rostral part of the proximal subiculum. Further caudally in the right hemisphere, retrograde cell body label was more diffuse and predominantly located in the distal subiculum (Fig. 4C). An appreciable number of retrogradely labeled cells was present in the postsubiculum bilaterally, consistent with uptake by the anterodorsal thalamic nucleus (Figs. 4B,C). A dense plexus of retrogradely labeled cells was present in the ventral subiculum of the left hemisphere, corresponding to the hemisphere with an intact fornix. In the right ventral subiculum, the hemisphere in which the lateral half of the fornix was destroyed, no retrograde cell body label was present.

### Group 3—Anteroventral Thalamic Nucleus Injections With Fornix Lesions (Cases JAR182_1 and 2)

In both cases, bilateral fornix lesions were made (Fig. 2), in combination with Fast Blue tracer injections that targeted the anteroventral thalamic nucleus (right hemisphere in both cases). Additionally, in case JAR182_1, multiple injections of Fluororuby were made into the left dorsal subiculum and multiple injections of BDA into the right dorsal subiculum, while in case JAR182_2, multiple bilateral injections of Fluororuby were made into the dorsal subiculum.

**Figure 5:**
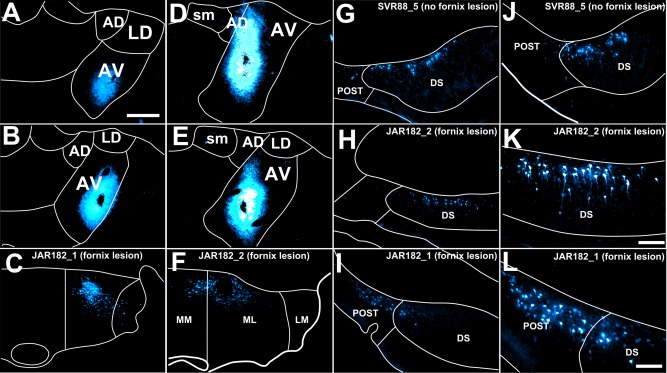
Fluorescent photomicrographs showing two cases (JAR182_1 and JAR182_2) that received bilateral fornix lesions combined with injections of Fast Blue centered in the anteroventral thalamic nucleus (A, B and D, E, respectively). One further control case received a near identical Fast Blue injection but without a fornix lesion (SVR88_5; G, J). The resulting retrograde label in the ipsilateral pars lateralis subdivision of the medial mammillary nucleus is also shown (JAR182_1, C; JAR182_2, F). G–J. Fluorescent photomicrographs showing appreciable retrograde label in the dorsal subiculum in control case SVR88_5 at both rostral (G) and caudal (J) subicular levels alongside much reduced retrograde label in dorsal subiculum of cases JAR182_2 (H, K) and JAR182_1 (I, L). Note that K and L show higher (20×) magnification photomicrographs of H and I, respectively. CC, corpus callosum; AD, anterodorsal thalamic nucleus; AV, anteroventral thalamic nucleus; DS, dorsal subiculum; LD, laterodorsal thalamic nucleus; LM, lateral mammillary nucleus; ML, pars lateralis of the medial mammillary nucleus; MM, pars medialis of the medial mammillary nucleus; POST, postsubiculum; sm, stria medullaris. Scale bar in photomicrographs A–J = 250 µm; K, L = 100 µm. [Color figure can be viewed in the online issue, which is available at wileyonlinelibrary.com.]

In case JAR182_1, the fornix lesion was substantial, without damage to the thalamus or the cingulum, and removed most of the fornix with the exception of some sparing in the dorsal‐most medial region and a narrow strip in the ventral most extent of the tract (Fig. 2). The Fast Blue injection site was centered in the anteroventral thalamic nucleus (Figs. 5A,B) and tracer uptake by the anteroventral thalamic nucleus was confirmed by retrogradely labeled cells in pars lateralis of the medial mammillary nucleus (Fig. 5C). Additional, sparse retrograde cell body label was present in the lateral mammillary nuclei, a likely result of tracer uptake by the anterodorsal thalamic nucleus. The absence of retrogradely labeled cells in pars medialis of the medial mammillary nucleus (Fig. 5C) was consistent with a lack of tracer uptake by the neighboring anteromedial thalamic nucleus.

Retrograde cell label was present in the rostral subiculum (<20 cells/section, ipsilateral to the injection; Figs. 5H,K), but was weaker relative to intact control cases involving virtually identical injections into the anteroventral thalamic nucleus (SVR88_5; Figs. 5G,J; 120 cells/section and SVR88_6; 80 cells/section). Further caudally, the number of retrogradely labeled cells in JAR182_1 was reduced (5–10 cells/section) and distributed entirely in the distal subiculum and on the border of the postsubiculum. Ipsilateral to the Fast Blue injection site there were ample retrogradely labeled cells in the postsubiculum, consistent with tracer uptake by the anterodorsal thalamic nucleus. No retrograde cell body label was present in the ventral subiculum.

In an equivalent case (JAR182_2), the fornix lesion was large but incomplete, with significant medial sparing (Fig. 2). In addition, the lesion resulted in slight, bilateral damage to the anterodorsal and anteroventral thalamic nuclei, although fiber tracts, including the stria terminalis, were not affected. The Fast Blue injection site was centered in the anteroventral thalamic nucleus (Figs. 5D,E). Fast Blue uptake by the anteroventral nucleus was confirmed by the presence of retrogradely labeled cells in pars lateralis of the medial mammillary nucleus (Fig. 5F). In addition, a very sparse population of retrogradely labeled cell bodies was present in pars medialis of the medial mammillary nucleus, corresponding to some tracer uptake by cells of the neighboring anteromedial thalamic nucleus.

Retrograde labeled cell bodies were present bilaterally in the rostral dorsal subiculum (30–40 cells/section; Figs. 5H,K) and confined, more or less, exclusively to distal regions. The number of labeled cells with the more caudal (i.e., intermediate) subiculum was greatly reduced (<5 cells/section). Retrogradely labeled cells were present in the deep layers of the ipsilateral postsubiculum, varying in density from 5 to 15 cells/section rostrally to 30 to 40 cells/section in more caudal regions. In contrast to case JAR182_1, retrogradely labeled cells were present in a localized rostral region of the ventral subiculum (20–30 cells/section) and were confined to the hemisphere ipsilateral to the Fast Blue injection into the anteroventral nucleus.

### Group 4—Laterodorsal Thalamic Nucleus Injections With Bilateral Fornix Lesions (Case SVR042_1)

In this case, the bilateral Fast Blue injection tracts passed through the caudal anteromedial thalamic nuclei to terminate in the region of the mammillothalamic tract. Consequently, the subiculum was almost completely devoid of Fast Blue labeled cells in either hemisphere, with just a handful of labeled cells in the postsubiculum. For this reason, attention focussed on the Diamidino Yellow injections. The left injection was centered within the laterodorsal nucleus, while the right Diamidino Yellow injection tract traversed the laterodorsal nucleus to position itself within the posterior thalamic nuclear group. The fornix lesion was extensive in both hemispheres (Fig. 2), resulting in additional bilateral damage to the uppermost parts of the anterodorsal and anteroventral thalamic nuclei, as well as to the stria medullaris, although the stria terminalis was spared. In addition, the lesion extended dorsally to reach the cingulum bundle, bilaterally. As expected, no retrogradely labeled Diamidino Yellow cells were present in the mammillary body nuclei of either hemisphere (laterodorsal thalamic nucleus injection).

Despite the fornix lesion, many cells labeled by the retrograde transport of Diamidino Yellow from the laterodorsal thalamic nuclei were found within the deep layers of the left rostral postsubiculum. Further caudally in the postsubiculum, the number of retrogradely labeled Diamidino Yellow cells diminished. The observed cell body label did not appear to be reduced relative to an equivalent control case (SVR75_9), albeit involving an injection of Fast Blue, rather than Diamidino Yellow, into the laterodorsal thalamic nucleus.

### Group 5—Postsubiculum Injections With Fornix Lesions (Case SVR78_28 and SVR87_6)

#### Injection sites and fornix lesion/anterograde label

Case SVR78_28 (described previously) also received an injection of WGA‐HRP, which was centered in the caudal part of the distal dorsal subiculum of the left hemisphere. From the injection site, it was possible to follow dense fiber label within the ipsilateral dorsal fornix, i.e., above the corpus callosum, as well as in the fimbria of the fornix (bilaterally) up to the lesion site within the fornix. Rostral to the lesion, labeled fibers were present ipsilaterally in the columns of the fornix, albeit markedly reduced in density when compared to a case with an intact fornix (e.g., case JAR178_1 (WGA‐HRP) or case JAR182_3 (BDA/Fluororuby)).

Anterograde thalamic label (fibers and terminals) in case SVR78_28 was present throughout the extent of the ipsilateral laterodorsal thalamic nucleus, seemingly being innervated along its lateral border by fibers that take a path via the internal capsule, before coursing medially to form a fiber tract situated beneath the stria terminalis. In addition, retrograde label, i.e., cell soma and fiber label, were present in the ipsilateral anterodorsal thalamic nucleus. Diffuse fiber label was also present in the dorsolateral anteroventral thalamic nucleus (ipsilaterally). No fiber label was present in the anteromedial thalamic nucleus in either hemisphere.

**Figure 6:**
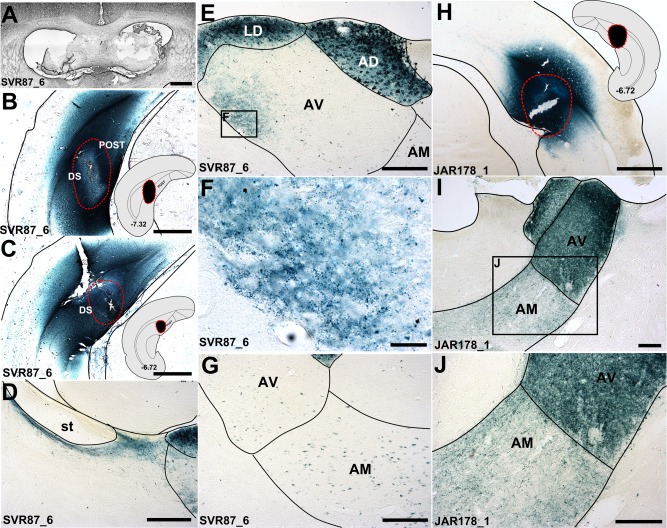
Brightfield photomicrographs showing the anterograde and retrograde transport of WGA‐HRP following injections into the dorsal subicular cortices, one combined with a fornix lesion (case SVR88_6; A) the other with an intact fornix (JAR178_1). B, C, H: WGA‐HRP injection sites into the subiculum/postsubiculum (B, C; SVR88_6; H; JAR178_1). The insets show where the injections were centered schematically and their position along the anterior posterior axis (coordinates correspond to Paxinos and Watson, 2005). D: labeled fibers coursing beneath the stria terminalis in case SVR88_6, corresponding to the nonfornical pathway described by Meibach and Seigel (1977b); E, F: Low (E; 10×) and high (F; 40×) magnification photomicrographs showing WGA‐HRP fiber and terminal label in the dorsolateral third of the anteroventral thalamic nucleus fiber, alongside much dense soma and fiber label in the anterodorsal and laterodorsal thalamic nucleus (E), with only retrograde (cell soma) label in the remainder of the anteroventral thalamic nucleus and anteromedial nucleus (G); I, J: Dense, bilateral fiber and cell body label throughout the anterior thalamus, following bilateral injections of WGA‐HRP into the dorsal subiculum in a case with an intact fornix (JAR178_1). AD, anterodorsal thalamic nucleus; AM, anteromedial thalamic nucleus; AV, anteroventral thalamic nucleus; DS, dorsal subiculum; LD, laterodorsal thalamic nucleus; POST, postsubiculum; st, stria terminalis. Scale bars: A, B, C, and H = 1,000 µm; D, E, G, I and J = 250 µm; F = 50 µm. [Color figure can be viewed in the online issue, which is available at wileyonlinelibrary.com.]

Similarly, but with a complete fornix lesion (Fig. 2), case SVR87_6 received unilateral injections of WGA‐HRP into the distal dorsal subiculum and postsubiculum of the left hemisphere (Figs. 6B,C). Rostral to the injection, ipsilateral fibers were labeled in the dorsal fornix, with additional bilateral fiber label in the fimbria of the fornix. Rostral to the fornix lesions, consistent with the histological verification of the lesion, no fiber label was evident in the columns of the fornix or the postcommissural fornix, i.e., the tract disconnection appeared complete.

**Figure 7:**
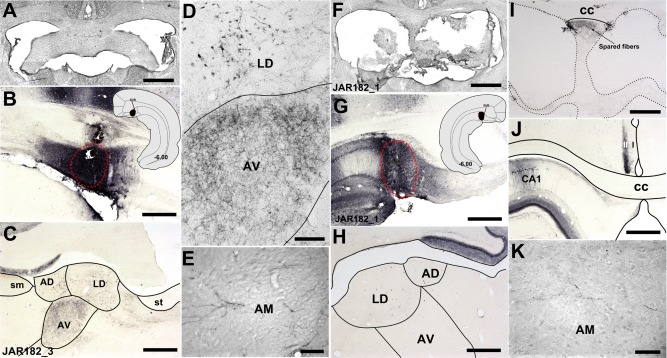
Brightfield photomicrographs showing anterograde and retrograde label following injections of BDA into the dorsal subiculum of a control case without a fornix lesion (JAR182_3; A–F) and a fornix lesion case (JAR182_1; F–K). The BDA injection sites were centered in the dorsal subiculum in both control (B) and lesion cases (G). Labeled anterograde fibers could be followed through the dorsal medial fornix to the anterior thalamus (C–E, H, K). In control case JAR182_3, anterograde label was present bilaterally in the anteroventral thalamic nucleus (C–E), and to a lesser extent in the anteromedial thalamic nucleus (E) and the laterodorsal thalamic nucleus (C, D). The fornix lesion in JAR182_1 (F) was extensive with only a narrow strip of medial sparing, however some intact labeled fibers could be followed through this region (I). Light anterograde and retrograde label was present in the laterodorsal thalamic nucleus (H), however, anterograde label in the anteroventral (H) and, at higher magnification (40×), in the anteromedial thalamic nuclei (K) was virtually absent. Dense ipsilateral anterograde label in layer two of the retrosplenial cortex provided evidence for effective tracer uptake and transport (J). AD, anterodorsal thalamic nucleus; AM, anteromedial thalamic nucleus; AV, anteroventral thalamic nucleus; CC, corpus callosum; CA1; hippocampal field CA1; DS, dorsal subiculum; LD, laterodorsal thalamic nucleus; sm, stria medullaris; st, stria terminalis; I, layer 1 of the retrosplenial cortex; II, layer 2 of the retrosplenial cortex. Scale bars in A–C, F–H, and J = 1,000 µm; D and I = 250 µm and E and K = 100 µm. [Color figure can be viewed in the online issue, which is available at wileyonlinelibrary.com.]

Dense fiber and cell body label were present in the ipsilateral anterodorsal thalamic nucleus (Fig. 6E). Dense fiber and terminal label was also present ipsilaterally in the laterodorsal thalamic nucleus (Fig. 6E). These two thalamic nuclei were innervated by labeled fibers of the internal capsule that coursed medially within a pathway situated beneath the stria terminalis (Fig. 6D). Retrograde cell body label was present in the anteroventral (Figs. 6E,F) and anteromedial (Fig. 6G) thalamic nuclei, both confined to the ipsilateral hemisphere. This retrograde cell body label was consistent with the presence of labeled fibers in the cingulum bundle. No fiber or terminals were observed in the anteromedial thalamic nucleus, however a dense localized patch of label was present within the dorsolateral part of the anteroventral thalamic nucleus (Figs. 6E,F). This label appeared to involve anterograde termination, though the retrograde label in other parts of the anterior thalamic nuclei meant that this same fiber label could, at least in part, include efferent fibers from the anteromedial and anteroventral thalamic nuclei that join the cingulum, before turning caudally to reach the hippocampus (see Domesick, 1970; Shibata, 1993). In equivalent cases with injections of BDA (JAR182_3 (Figs. 7A–E) and JAR182_4) or WGA‐HRP (JAR178_1; Figs. 6H–I) into the dorsal subiculum but where the fornix was left intact, in addition to (fornical) anterograde input to the anteromedial and anteroventral thalamic nuclei, the anterodorsal and laterodorsal thalamic nuclei were, again, seemingly innervated by nonfornical fibers passing beneath the stria terminalis. In addition, fibers from the same nonfornical pathway appeared to extend to the dorsal lateral part of the anteroventral thalamic nucleus, consistent with a partial nonfornical input to this nucleus.

### Group 6—Dorsal Subiculum Injections With Fornix Lesions (Cases (JAR182_1, JAR182_2)

Anterograde tracer injections (BDA and Fluororuby) into the dorsal subiculum (Fig. 7G) of case JAR182_1 resulted in dense dorsal fornix and commissural fiber label that could be followed rostrally to the level of the fornix lesion. Intact fibers in the spared dorsomedial region of fornix (Fig. 7I), described above (see Group 3), could be followed to the level of the medial septum. However, while these fibers descended to join the postcommissural fornix, there was virtually no anterograde fiber label in the anterior thalamus (Fig. 7H, excluding a handful of fibers and terminals in the anteromedial thalamic nucleus (Fig. 7K).

Case JAR182_2 also received Fluororuby injections into the dorsal subiculum. Spared anterograde fibers could be followed rostral to the fornix lesion before descending to form the postcommissural fornix. Within the anterior thalamus, diffuse fiber label was present in the anteroventral and anteromedial thalamic nuclei. This label was appreciably lighter when compared with the anterograde label observed in the two intact control cases in which Fluororuby was injected into the dorsal subiculum (cases JAR182, 3 and JAR182_4). In the two control cases JAR182_3 (Fig. 7A) and JAR182_4, in addition to the Fluororuby injections into the left hemisphere, BDA injections were made into the dorsal subiculum of the right hemisphere (Fig. 7B). These injections resulted in dense bilateral anterograde fiber label in the anteroventral (Figs. 7C,D) and, to a lesser degree, the anteromedial thalamic nuclei (Fig. 7E). In an additional control case (i.e., with an intact fornix; JAR178_1) that received bilateral injections of WGA‐HRP into the dorsal subiculum, very dense fiber and cell body label (bilaterally) was present in the anteroventral and anteromedial thalamic nuclei, as well as in the anterodorsal (Figs. 6I,J) and laterodorsal thalamic nuclei.

## DISCUSSION

Across a range of species, the anterior thalamic nuclei receive dense inputs from the subicular cortices, rather than the hippocampus proper (e.g., Meibach and Siegel, 1975a,b; Swanson and Cowan, 1975; Rosene and Van Hoesen, 1977; Krayniak et al., 1979). There are, however, species differences concerning the extent of those direct connections that involve the fornix (Valenstein and Nauta, 1959). In monkeys, the overwhelming majority of the projections to the anterior thalamic nuclei (anteromedial, anteroventral, and anterodorsal nuclei) connect via the postcommissural fornix (Poletti and Cresswell, 1977; Aggleton et al., 1986). In contrast, the laterodorsal thalamic nucleus is jointly supplied by fornical and nonfornical (via the pulvinar) pathways in macaques (Fig. 1C; Aggleton et al., 1986; Saunders et al., 2005), with a possible very light, additional projection to the anterodorsal nucleus, which also relies on the temporopulvinar pathway (Aggleton, 2008). The present study examined the corresponding routes in the rat.

Previous studies agree that the projections from the rat subiculum to the anteroventral nucleus largely involve the postcommissural fornix (Meibach and Siegel, 1977b; Swanson and Cowan, 1977), but there is disagreement over the principal route to the anteromedial nucleus (Meibach and Siegel, 1977b; Swanson et al., 1987). Using intact rats, Meibach and Siegel (1977b) described a split, whereby the distal subiculum relies on the fornix to reach the anteroventral nucleus while the proximal subiculum (nearest to CA1) joins the internal capsule to reach the anteromedial nucleus (Meibach and Siegel, 1977b; see Fig. 1). The presence of fornix fibers that terminate in the anteromedial nucleus could not, however, be excluded due to technical limitations (Meibach and Siegel, 1977b). In response, a subsequent study visualised subicular‐anterior thalamic projections after cutting postcommissural fornix fibers (Davis and Kent, 1979). This method confirmed that some projections from the dorsal subiculum to the anterior thalamic nuclei rely on fornical fibers, but the extent to which this applies to the anteromedial thalamic inputs remained undetermined (Davis and Kent, 1979).

The present study also combined fornix lesions with anterior thalamic tracer injections (see Davis and Kent, 1979), but with a deliberate emphasis on placing retrograde tracers into the anteromedial nucleus. Additional approaches involved injecting anterograde tracers (WGA‐HRP, BDA or Fluororuby) into the dorsal subiculum, again in rats with fornix lesions. Although WGA‐HRP has the potential limitation that it is transported both retrogradely and anterogradely (e.g. Gonatas et al., 1979; Itaya, 1987), it has the advantage that fiber pathways are readily visualized. In practice, the retrograde transport of WGA‐HRP caused few issues of interpretation as the results with this marker were supported by the additional anterograde tracers employed, as well as the separate retrograde tracer studies.

In none of the present cases was there evidence of anything other than a light nonfornical route from the subiculum to the anteromedial thalamic nucleus (Fig. 1B), comprising at most 10% of the inputs. In fact, some cases with fornix lesions showed a complete lack of labeled cells in the subiculum following the injection of retrograde tracers into the anteromedial thalamus. In other cases there was a residual pattern of retrograde label in the rostral dorsal subiculum (Figs. 4E and 5H). The number of labeled cells was always substantially less than when the fornix was largely spared (Fig. 4B) though, with even modest tract sparing, the number of labeled cells increased appreciably (e.g. Fig. 4G). These findings are supported by those cases with anterograde tracer (WGA‐HRP, Fluororuby and BDA) injections into the dorsal subiculum combined with lesions of the fornix (Figs. 6 and 7). Here, little or no anterograde terminal label was seen in the anteromedial thalamic nucleus. In contrast, retrogradely labeled neurons were present in the anteromedial nucleus when WGA‐HRP was injected into the subiculum, reflecting the direct anterior thalamic projections to the hippocampal formation, which use a spared pathway, the cingulum (Domesick, 1970; Shibata, 1993; Figs. 7E,F). The absence of retrograde label in the anteromedial nucleus following BDA and Fluororuby injections into the dorsal subiculum reflects how these tracers are predominantly transported anterogradely.

The present study found evidence for nonfornical projections to the thalamus from the postsubiculum (see also van Groen and Wyss, 1990b). Levels of postsubiculum label following retrograde tracer injections into the laterodorsal thalamic nucleus seemed largely unaffected by fornix lesions. In addition, when targeting the anteromedial thalamic nucleus, presumed unintentional retrograde tracer uptake by the anterodorsal thalamic nucleus resulted in postsubicular label in rats with fornix lesions (see van Groen and Wyss, 1990b; Yoder and Taube, 2011). The anterograde tracer cases provided further evidence for nonfornical inputs to the anterodorsal and laterodorsal thalamic nuclei (Fig. 6). Nonfornical projections from the postsubiculum passed below the stria terminalis before turning medially to reach both the laterodorsal and anterodorsal thalamic nuclei (e.g. Fig. 6D; see also Swanson and Cowan, 1977, van Groen and Wyss, 1990a,b). In addition, the dorsal third of the anteroventral thalamic nucleus appears to receive light inputs from the postsubiculum and the distal subiculum, using this same nonfornical route (van Groen and Wyss, 1990a,b). In the present study, nonfornical label was seen in restricted parts of the dorsolateral part of the anteroventral nucleus (Figs. 6E,F). The evidence for both fornical and nonfornical inputs to the anteroventral thalamic nucleus also comes from retrograde tracer injections specifically targeting this nucleus (Fig. 5). Retrograde postsubicular and distal subiculum cell label was present irrespective of fornix lesions (Fig. 5). Finally, ventral subiculum inputs to the anterior thalamus appear to be exclusively fornical. In those cases with extensive fornix lesions, no retrograde label was present in the ventral subiculum, while in those cases where the fornix was intact or had appreciable sparing, ventral subiculum retrograde label was only present in the hemisphere ipsilateral to the Fast Blue injection site.

Before concluding that the hippocampal inputs to the anteromedial thalamic nucleus primarily rely on the fornix, it is important to consider several issues. The consistent presence of label in the medial mammillary nucleus confirmed the efficacy of the tracers, as well as providing additional evidence for anteromedial nucleus involvement (e.g., Figs. 3D,G). A potential concern is that the extensive lesions required to transect the fornix may have damaged the adjacent dorsal thalamus, including the routes taken by nonfornical fibers (e.g., Fig. 3B). In fact, very evident losses of subicular label after anteromedial nucleus injections were still seen in cases with fornix lesions but little or no additional thalamic damage. Even when extra‐fornical damage was observed, it typically did not reach the stria terminalis, i.e., it was far away from the internal capsule route depicted by Meibach and Siegel (1977b, Fig. 1A). This conclusion is supported by the ample retrosplenial label (unreported) found in those cases with additional thalamic damage, thereby providing a measure of both tracer efficacy and the integrity of the internal capsule when the lesion extended beyond the fornix (van Groen and Wyss, 1992).

A very different concern is whether cutting the fornix causes retrograde changes in the subiculum leading to a loss of signal. This possibility can be discounted as there was no evidence of retrograde hippocampal degeneration (see also Daitz and Powell, 1954). Indeed, subicular fibers still transported WGA‐HRP up to the site of the fornix lesion (e.g., case SVR87_6, see also Aggleton et al., 1986). Furthermore, very few individual neurons in the subiculum terminate in both the anteromedial and anteroventral nuclei (Wright et al., 2013). The latter point is important as, if such bifurcating neurons exist, they could explain how a lesion affecting one pathway might unintentionally affect another pathway.

The question remains as to why Meibach and Siegel (1977b) reported such different findings. One factor concerns the nature of the thalamic pathway from the postcommissural fornix. From the descending column into the thalamus there is no discrete pathway; instead, the fornix fibers fan out diffusely to reach their targets. For this reason, any such fiber label would be difficult to detect on coronal sections, a point acknowledged by Meibach and Siegel (1977b). Consequently, it was necessary to include cases with fornix lesions.

The present findings point to clear commonalities between rats and monkeys (Fig. 1). One of the few species differences is that, while there may only be an extremely light nonfornical input in the macaque brain to the anterodorsal nucleus (Aggleton, 2008), the rat anterodorsal thalamic input appears disproportionately reliant on nonfornical pathways (van Groen and Wyss, 1990a,b). In the rat brain, the alternate (nonfornical) pathways principally involve the projections from the postsubiculum to the anterodorsal and laterodorsal nuclei (van Groen and Wyss, 1990a,b; Yoder and Taube, 2011). All three sites contain head direction cells, i.e., cells that preferentially fire when the rat is facing in a particular direction (Taube, 2007). Their apparent reliance on the internal capsule pathway appears, once again, to mark out this navigation system as having distinct anatomical properties (Hopkins, 2007).

The fornix also provides the route for all of the hippocampal projections to the mammillary bodies in both rats and monkeys (Swanson and Cowan, 1975, 1977; Aggleton et al., 2005). For these reasons, it is to be expected that fornix lesions will disconnect many of the direct and indirect influences of the hippocampal formation on the anterior thalamic nuclei (Vann et al., 2000), with marked consequences for cognition. It is, therefore, unsurprising that the behavioral effects of fornix lesions in rats often mimic those of anterior thalamic lesions (e.g., Sutherland and Rodriguez, 1989; Aggleton et al., 1991, 1995; Warburton et al., 2000; Gaffan et al., 2001).

In light of the present findings, particular interest focuses on those occasions when anterior thalamic lesions appear more disruptive than fornix lesions, as such instances may reflect the contributions of nonfornical hippocampal pathways. These examples include the learning of spatial biconditional tasks and fixed place learning in the Morris water‐maze (Sziklas and Petrides, 1999, 2002; Warburton and Aggleton, 1999; Aggleton et al., 2009; but see Sutherland and Rodriguez, 1989). These findings suggest that nonfornical pathways from the subicular cortices to the anterior thalamus can aid spatial learning that involves a response rule to a set location (Dumont et al., 2010, 2014). In contrast, the fornical routes to the anterior thalamus may be especially important for spatial tasks that involve differential responses to multiple locations when there is high proactive interference, e.g., T‐maze alternation and delayed nonmatching‐to‐position (Warburton and Aggleton, 1999; Warburton et al., 2000; Aggleton et al., 1991, 1995, 2009; Dumont et al., 2014). This tentative functional division highlights the importance of understanding the balance between the fornical and nonfornical routes from the hippocampal formation to the anterior thalamic nuclei.
